# Supplementary material for: The upper respiratory tract microbiome of indigenous Orang Asli in north-eastern Peninsular Malaysia
Source: NPJ Biofilms Microbiomes. 2021 Jan 5;7:1. doi: 10.1038/s41522-020-00173-5 (PMC7785749; doi:10.1038/s41522-020-00173-5)
Supplement: Supplementary file 1 — Supplementary Information [file 41522_2020_173_MOESM1_ESM.pdf]

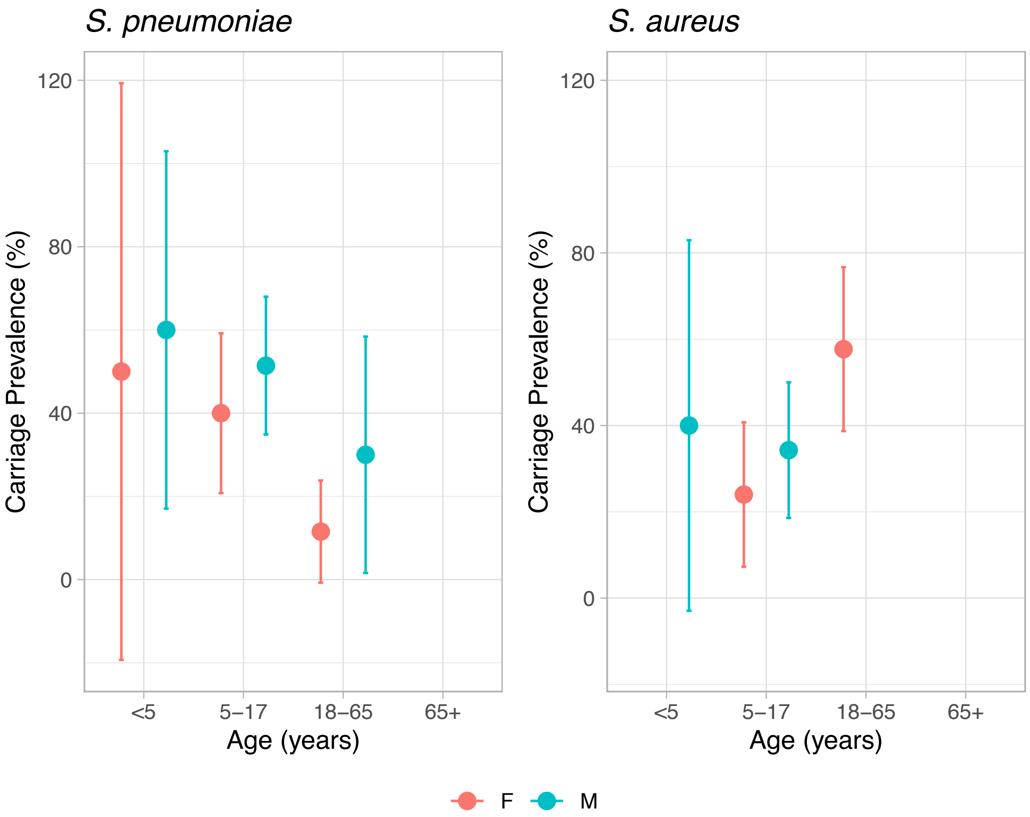

Supplementary Figure 1: Carriage Prevalence of *S. pneumoniae* and *S. aureus* in Female and Male Study Participants. Point estimates for percentage carriage prevalence of *S. pneumoniae* and *S. aureus* are shown across three age groups <5, 5 to 17, 18 to 65 and 65+. Missing data indicates either carriage was not absent, not determined, or gender was not recorded. Carriage was estimated overall i.e. a positive swab culture from either nasal or nasopharyngeal swab. Error bars show 95% CI intervals.

Supplementary Table 1: Multi- versus single-species carriage of *Streptococcus pneumoniae* (Spn), *Staphylococcus aureus* (Sa), *Haemophilus influenzae* (Hi) and *Moraxella catarrhalis* (Mc). Forty-seven individuals were culture negative. Seven had single-species profiles involving *Klebsiella pneumoniae* (n=3), α-haemolytic *Streptococci* (n=2) or *Pseudomonas aeruginosa* (n=2). A final thirteen had different multi-species profiles to those listed below.

Bacterial Species	N	%
Sa	25	19.23
Spn	16	12.31
Spn-Hi	7	5.38
Spn-Sa	5	3.85
Hi	2	1.54
Mc	1	0.77
Spn-Mc	1	0.77
Hi-Sa	1	0.77
Hi-Mc	1	0.77
Spn-Hi-Sa	1	0.77
Spn-Hi-Mc	1	0.77
Sa-Mc	0	0.00
Hi-Sa-Mc	0	0.00
Sa-Mc-Spn	0	0.00
Sa-Mc-Spn-Hi	0	0.00

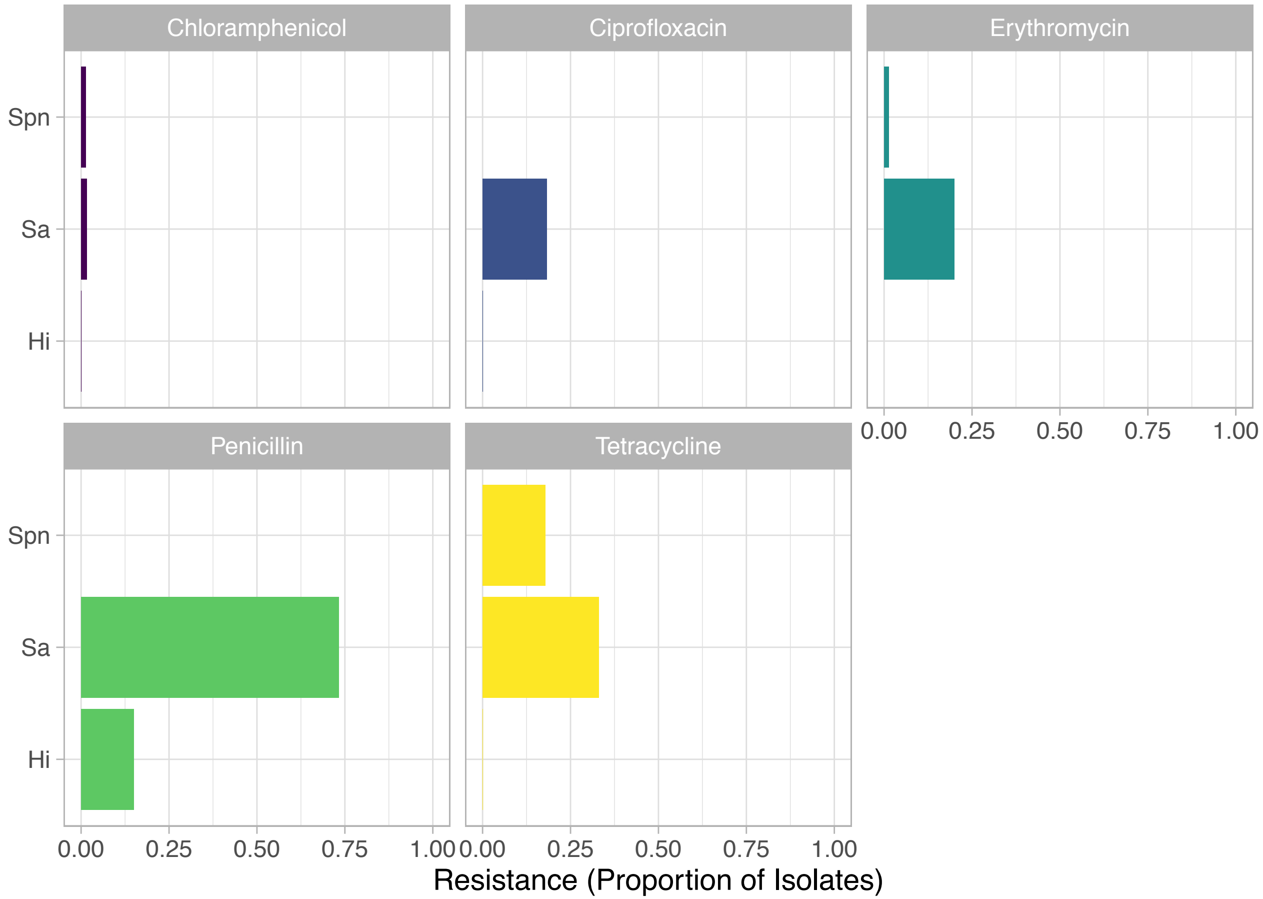

Supplementary Figure 2: Antibiotic resistance of isolates of *S. pneumoniae* (Spn; n=67), *S. aureus* (Sa; n=60) and *H. influenzae* (Hi; n=10). Columns represent the proportion of isolates that were resistant to tetracycline, chloramphenicol, erythromycin, ciprofloxacin and penicillin. Forty-four (73.3%) *S. aureus* isolates were resistant to penicillin, 20 (33.0%) to tetracycline, 12 (20%) to erythromycin and 11 (18.3%) to ciprofloxacin. Of the *H. influenzae* isolates three (15%) were resistant to penicillin. Twelve *S. pneumoniae* (17.9%) were resistant to tetracycline.

Genus	Frequency
NA	1887
Prevotella	118
Treponema	96
Leptotrichia	70
Corynebacterium	64
Oscillospira	63
Coprococcus	54
Ruminococcus	52
Blautia	49
Capnocytophaga	45
Deinococcus	37
Bacteroides	36
Clostridium	35
Fusobacterium	32
Actinomyces	29
Sphingomonas	29
[Prevotella]	28
Selenomonas	28
Acinetobacter	27
Moraxella	27
Streptococcus	26
Anaerococcus	24
Faecalibacterium	24
Nesterenkonia	24
Dorea	23
Lactobacillus	23
[Ruminococcus]	22
Neisseria	22
Porphyromonas	22
Veillonella	22
Alloiococcus	19
Haemophilus	19
Nocardioides	17
Pseudomonas	17
Aggregatibacter	16
Kaistobacter	16
Leptolyngbya	16
Campylobacter	15
Chryseobacterium	14
Parabacteroides	14
Paracoccus	14
Mycobacterium	13
Rubrobacter	13
[Eubacterium]	12
Dialister	12
Methylobacterium	12
Roseburia	12
Staphylococcus	12

Genus	Frequency
Kingella	11
Paenibacillus	11
Peptoniphilus	11
Schwartzia	11
Sutterella	11
Tannerella	11
Lachnospira	10
Megasphaera	10
Microbacterium	10
Rhodoplanes	10
Rothia	10
Stenotrophomonas	10
Actinobacillus	9
Alicyclobacillus	9
Bacillus	9
Bifidobacterium	9
Brevibacterium	9
Brevundimonas	9
Butyricimonas	9
Eikenella	9
Flavisolibacter	9
Actinomycetospora	8
Catonella	8
Devosia	8
Enhydrobacter	8
Flavobacterium	8
Gemmata	8
Micrococcus	8
Rhodobacter	8
Roseomonas	8
Arthrobacter	7
Atopobium	7
Chloronema	7
Lachnoanaerobaculum	7
Mogibacterium	7
Acholeplasma	6
Amaricoccus	6
Halomonas	6
Hymenobacter	6
Kocuria	6
Lysobacter	6
Marmoricola	6
Moryella	6
Mycoplasma	6
Parvimonas	6
Phenylobacterium	6
Propionibacterium	6
Pseudonocardia	6

Genus	Frequency
Rubellimicrobium	6
Rubrivivax	6
Saccharopolyspora	6
Salinicoccus	6
Scardovia	6
Sphingobacterium	6
Adlercreutzia	5
Aeromicrobium	5
Alistipes	5
Anaerostipes	5
Balneimonas	5
Cardiobacterium	5
Dermacoccus	5
Desulfovibrio	5
Facklamia	5
Halalkalicoccus	5
Kytococcus	5
Massilia	5
Oribacterium	5
Peptococcus	5
Propionicimonas	5
Rhodococcus	5
Riemerella	5
Shuttleworthia	5
Slackia	5
Streptomyces	5
Bdellovibrio	4
Bilophila	4
Brachybacterium	4
Bulleidia	4
Calothrix	4
Collinsella	4
Coprobacillus	4
Dermabacter	4
Dietzia	4
Gemmiger	4
Geodermatophilus	4
Granulicatella	4
Halococcus	4
Jeotgalicoccus	4
Lautropia	4
Leucobacter	4
Leuconostoc	4
Microlunatus	4
Odoribacter	4
Paraprevotella	4
Pimelobacter	4
rc4-4	4

Genus	Frequency
Rheinheimera	4
TG5	4
Thermomonas	4
Truepera	4
Variovorax	4
Achromobacter	3
Actinoplanes	3
Aerococcus	3
Agrobacterium	3
Akkermansia	3
Alishewanella	3
Anaerofustis	3
Beijerinckia	3
Bradyrhizobium	3
Butyricicoccus	3
Candidatus_Solibacter	3
Cellulomonas	3
Cellvibrio	3
Chroococcidiopsis	3
Cystobacter	3
Dechloromonas	3
Gallicola	3
Holdemania	3
Hydrogenophaga	3
Lactococcus	3
Macrococcus	3
Mesorhizobium	3
Methanobrevibacter	3
Nitrospira	3
Novosphingobium	3
Ochrobactrum	3
Olsenella	3
ph2	3
Phascolarctobacterium	3
Phycicoccus	3
Planctomyces	3
Prauserella	3
Pseudoramibacter	3
Pseudoxanthomonas	3
Scytonema	3
Sharpea	3
Shewanella	3
Tessaracoccus	3
Turicibacter	3
Varibaculum	3
Vogesella	3
Wautersiella	3
[Clostridium]	2

Acidaminococcus	2
Agrococcus	2
Agromyces	2
Anaeromyxobacter	2
Arsenicicoccus	2
Asplenium	2
Asteroleplasma	2
Azospirillum	2
Blvii28	2
Burkholderia	2
Butyrivibrio	2
Candidatus_Koribacter	2
Caulobacter	2
Chelativorans	2
Christensenella	2
Citricoccus	2
Cloacibacterium	2
Cohnella	2
Comamonas	2
Cupriavidus	2
Delftia	2
Eggerthella	2
Exiguobacterium	2
Friedmanniella	2
Gardnerella	2
Gemella	2
Gordonia	2
Granulicella	2
Helcococcus	2
Hyphomicrobium	2
Luteolibacter	2
Meiothermus	2
Methylibium	2
Mycoplana	2
Myroides	2
Oxalobacter	2
Pelomonas	2
Peptostreptococcus	2
Perlucidibaca	2
Planktothrix	2
Pleomorphomonas	2
Psychrobacter	2
Pyramidobacter	2
Ralstonia	2
Rhizobium	2
Sediminibacterium	2
Shinella	2
SMB53	2

Sphingopyxis	2
Spirosoma	2
Thermicanus	2
Williamsia	2
Xylanimicrob	2
Abiotrophia	1
Acidovorax	1
Actinocoralli	1
Actinomyceta	1
Adhaeribacte	1
Aeromonas	1
Alkanindiges	1
Anaerofilum	1
Anaerolinea	1
Anaerosinus	1
Anaerospora	1
Anaerotrunc	1
Anoxybacillu	1
Aquabacteriu	1
Aquamicrobi	1
Arcobacter	1
Arenimonas	1
Arthronema	1
Auritibacter	1
Azohydromo	1
Blastococcus	1
Bosea	1
Brachymona	1
Brasilonema	1
Brevibacillus	1
Caldilinea	1
Candidatus_I	1
Candidatus_I	1
Candidatus_I	1
Candidatus_)	1
Catenibacter	1
cc115	1
Cellulosimicr	1
Cetobacteriu	1
Chlamydia	1
Chryseomicr	1
Citrobacter	1
Conexibacter	1
Couchioplane	1
Cryocola	1
Curvibacter	1
Cylindrosperi	1
DA101	1

Genus	Frequency
Defluviibacter	1
Deinobacterium	1
Dermatophilus	1
Desulfobulbus	1
Desulfomicrobium	1
Dokdonella	1
Duganella	1
Dyadobacter	1
Dyella	1
Dysgonomonas	1
Edaphobacter	1
Edwardsiella	1
Enterococcus	1
Epulopiscium	1
Erythromicrobium	1
Filifactor	1
Fimbriimonas	1
Finegoldia	1
Flectobacillus	1
Fluviicola	1
Frankia	1
G07	1
Gemmatimonas	1
Geobacillus	1
Geobacter	1
Geomicrobium	1
Gloeocapsopsis	1
Gracilibacillus	1
Gulbenkiania	1
GW-34	1
Haliangium	1
Herbaspirillum	1
Herminiimonas	1
human	1
Hydrogenophilus	1
Hylemonella	1
Iamia	1
Janibacter	1
Janthinobacterium	1
Johnsonella	1
Lachnobacterium	1
Lacibacter	1
Lactonifactor	1
Leptothrix	1
Limnohabitans	1
Longispora	1
Luteimonas	1
Luteococcus	1

Genus	Frequency
Magnetospirillum	1
Marinococcus	1
Martelella	1
Megamonas	1
Methanobacterium	1
Methylomicrobium	1
Millisia	1
Mobiluncus	1
Morganella	1
Mucilaginibacter	1
Myxococcus	1
Nannocystis	1
Nevskia	1
Niabella	1
Nocardia	1
Novispirillum	1
Olivibacter	1
Opitutus	1
Ornithinimicrobium	1
p-75-a5	1
Parapedobacter	1
Parasegitibacter	1
Pedobacter	1
Petrobacter	1
Phaeospirillum	1
Phormidium	1
Photobacterium	1
Phyllobacterium	1
Pigmentiphaga	1
Pirellula	1
planctomycete	1
Pontibacter	1
Proteus	1
Providencia	1
Pseudanabaena	1
Pseudoclavibacter	1
Quadrisphaera	1
Ramlibacter	1
RFN20	1
Rhodocytophaga	1
Rickettsiella	1
Rikenella	1
Robinsoniella	1
Rubricoccus	1
Ruminofilibacter	1
Saccharomonospora	1
Saccharothrix	1
Salinibacterium	1

Genus	Frequency
Salinispora	1
Sandaracinobacter	1
Schumannella	1
Sebaldella	1
Segetibacter	1
Serinicoccus	1
SHD-231	1
Shimazuella	1
Silanimonas	1
Sinomonas	1
Skermanella	1
Smaragdicoccus	1
Solirubrobacter	1
Sphaerisporangium	1
Sphaerochaeta	1
Sphingobium	1
Sporosarcina	1
Stella	1
Steroidobacter	1
Subdoligranulum	1
Succinatimonas	1
Succinivibrio	1
Syntrophomonas	1
Tatumella	1
Telluria	1
Tepidimonas	1
Terracoccus	1
Tetrasphaera	1
Thauera	1
Thermobispora	1
Trichococcus	1
Uliginosibacterium	1
vadinHB04	1
Vibrio	1
WAL1855D	1
Weeksella	1
Weissella	1
Wolbachia	1
Wolinella	1
Xanthomonas	1
Yonghaparkia	1
Zoogloea	1

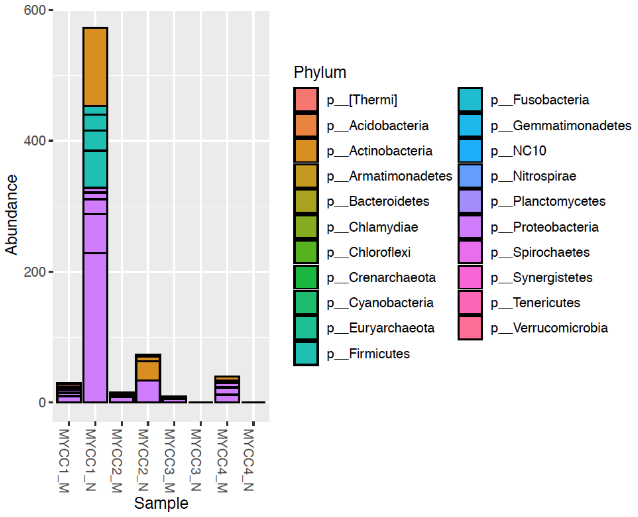

Supplementary Figure 3: Composition of control swab samples at Phylum and Genus level. Frequency indicates the cumulative frequency of ASVs with Genus X from the eight control samples.

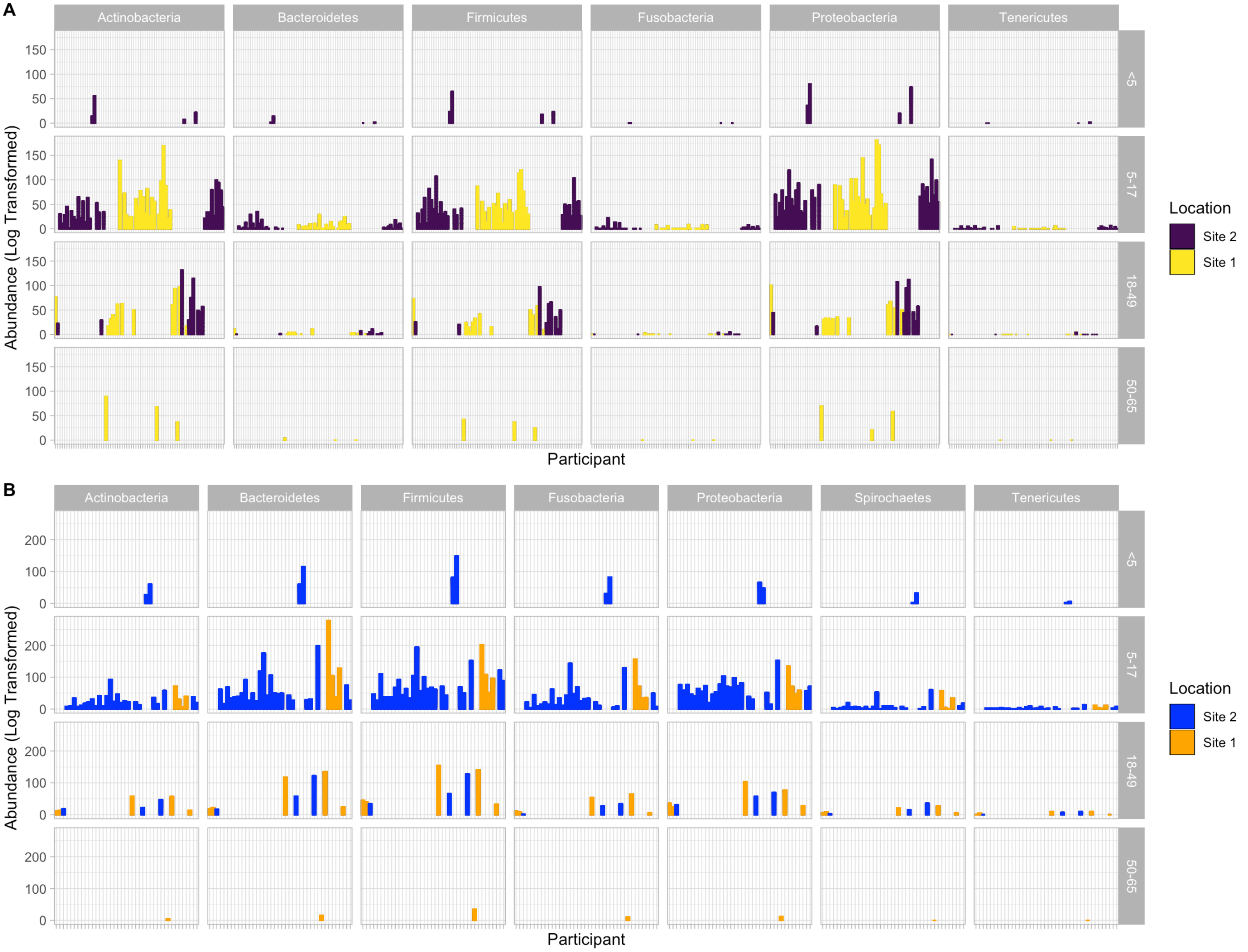

Supplementary Figure 4: Log Transformed Abundances of Phylum-level ASV Classifications in Nasal (top) and Oral (bottom) Samples. Bars are coloured by site of location. Firmicutes, Actinobacteria and Proteobacteria were the most abundant phyla in Nasal samples with Bacteroidetes, Firmicutes and Proteobacteria the most common in Oral samples. A clear increase in Firmicutes in nasal samples from Site 2 (Kampung Berua) can be seen.

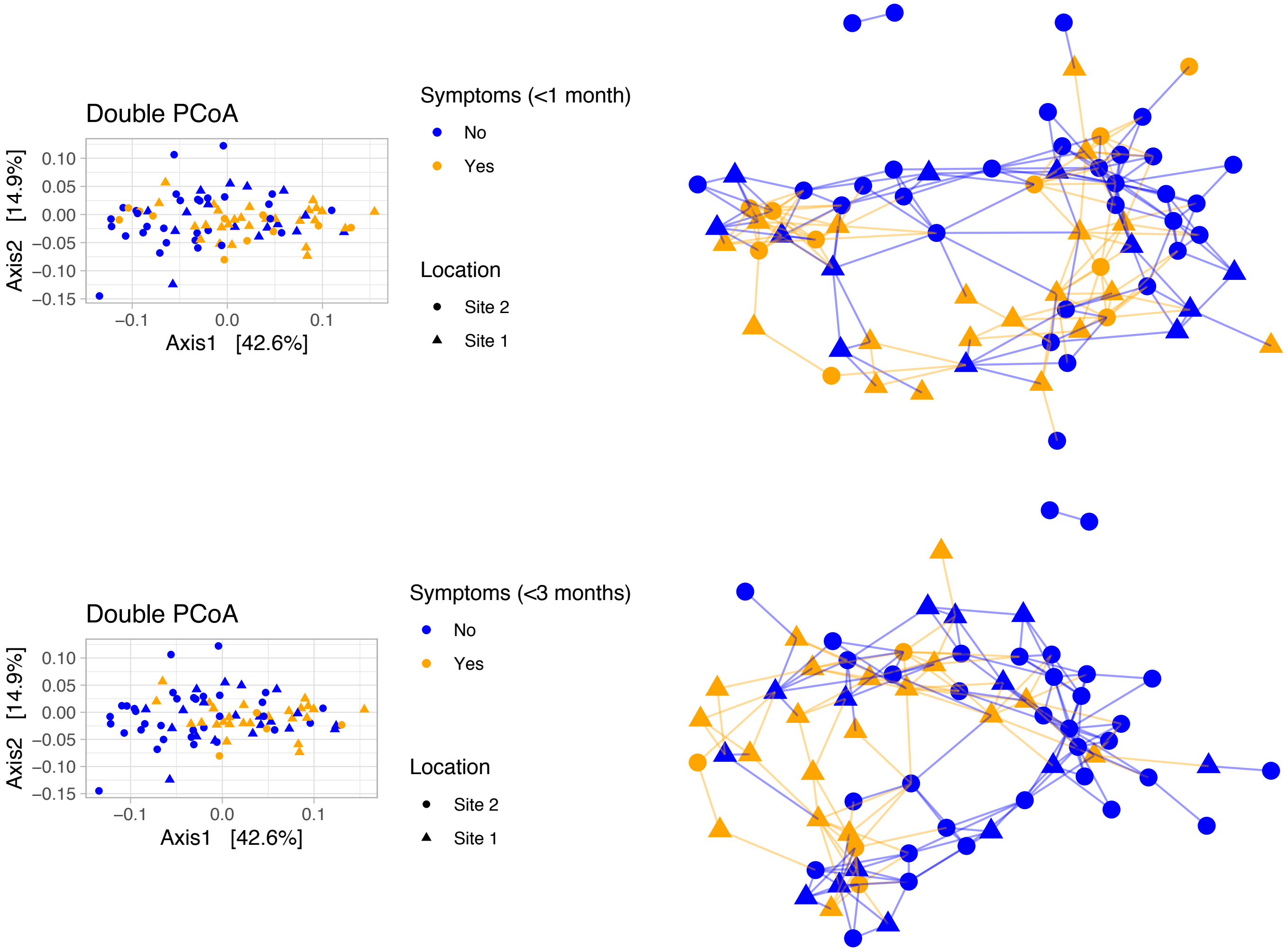

Supplementary Figure 5: Network and Double PCoA Beta-diversity plots comparing individuals that had reported respiratory symptoms within the last month (top) or within the last three months (bottom). Individuals reporting respiratory symptoms are shown in blue. Shape denotes the location with triangles Site 1 - Kampung Sungai Pergam and circles being Site 2 - Kampung Berua. Network construction was based on Jaccard distance with a maximum distance of 0.7.

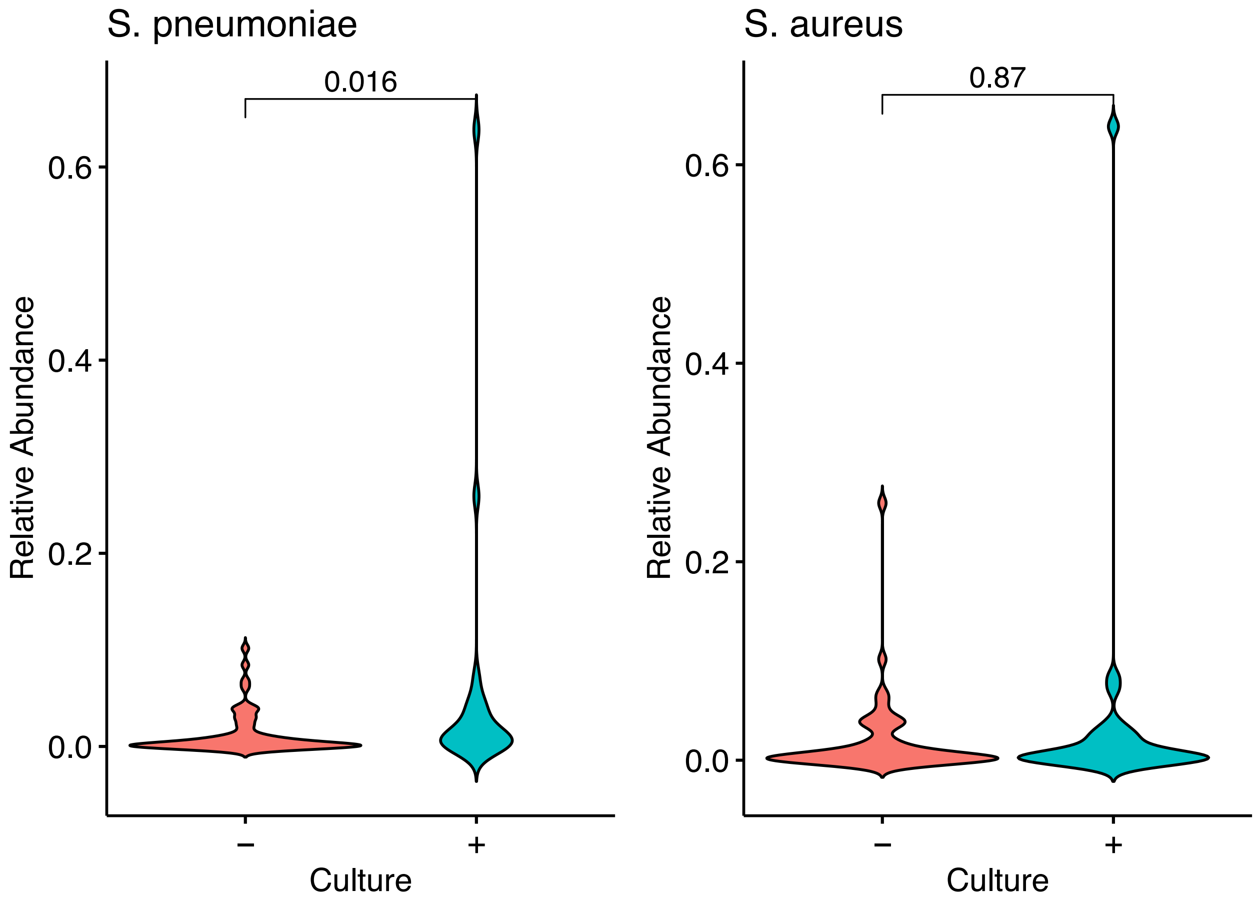

Supplementary Figure 6: Comparison of the relative abundance of Streptococcal and Staphylococcus ASVs between those that were culture positive or negative for *Streptococcus pneumoniae* or *Staphylococcus aureus*. *P* values were calculated using the nonparametric Wilcoxon test.

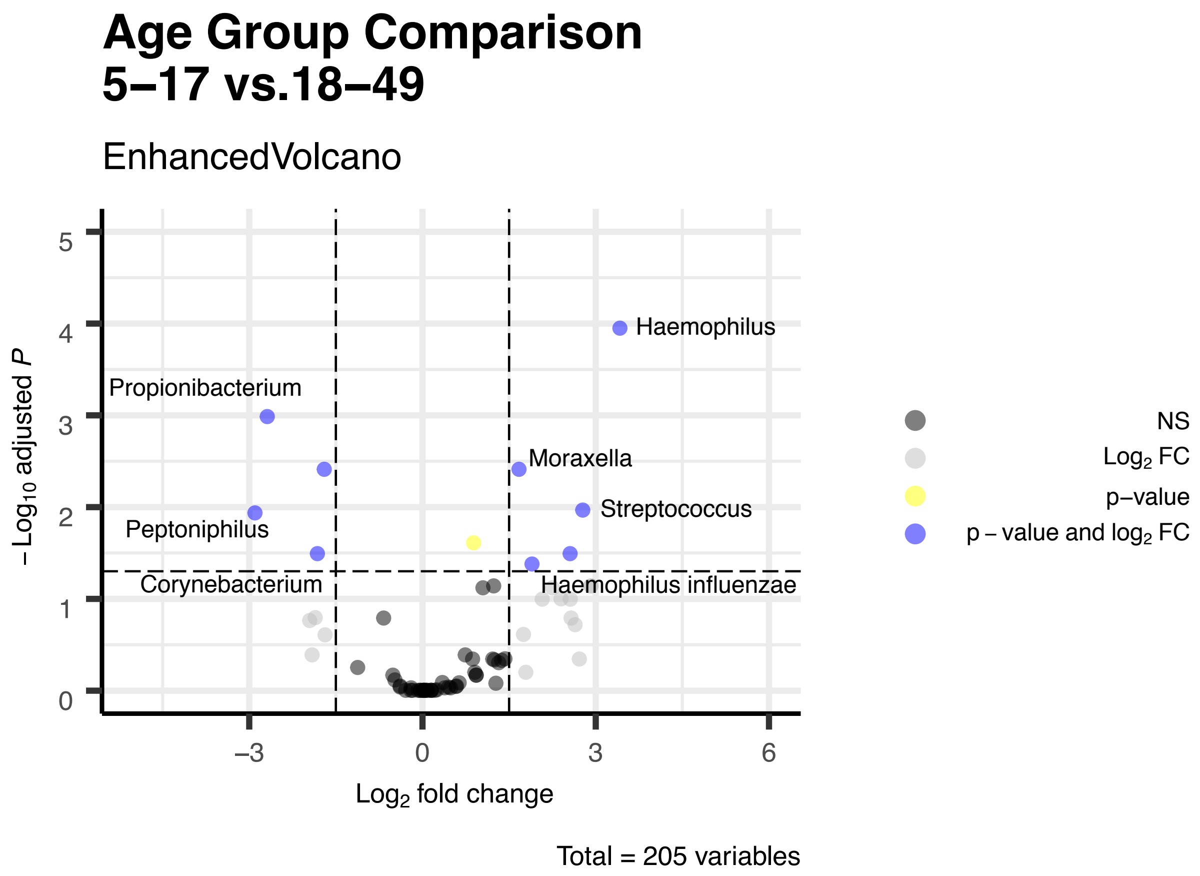

Supplementary Figure 7: Differentially Abundant ASVs Between Older Children (5-17 years) and Adults (18-49). Volcano plot showing the ASVs that were differentially abundant in comparisons between the two age groups. Here a p-value cut-off of 0.05 and a Log_2 fold change of 1.5 was applied. Those ASVs that pass both these thresholds are labelled where a genus and/or species taxonomic assignment was available. The right hand-side of the plot shows ASVs that had increased abundance in the 5-17 years olds compared to the 18-49. Conversely those to the left are those more abundant in the older age group.

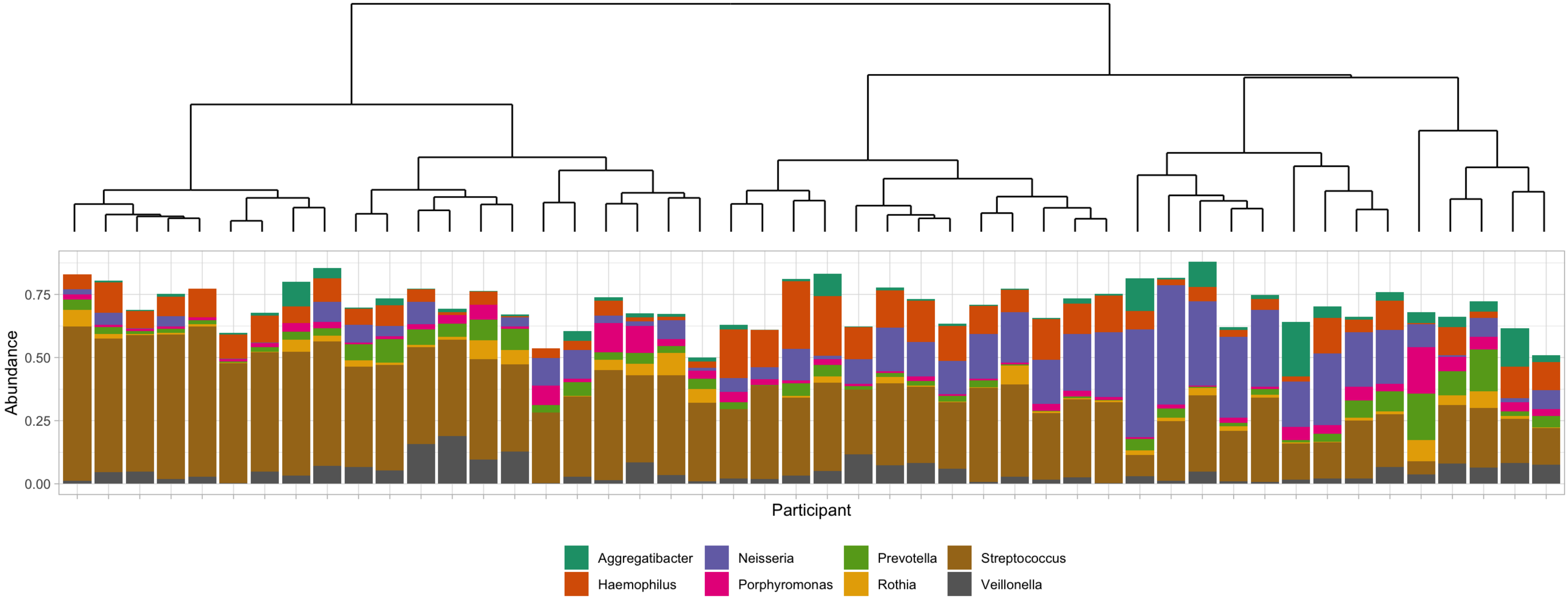

Supplementary Figure 8: Hierarchical Clustering of Oral Samples with Bray-Curtis Dissimilarity using Genus-level Relative Abundances. The dendrogram (top) shows clustering of samples with the below bar chart showing the relative abundance of the six most commonly observed Genera. Three genera are seen to be responsible for the majority of ASVs – *Streptococcus* (brown), *Haemophilus* (orange) and *Neisseria* (purple). Profiles characterised by an overabundance of *Streptococcus* or *Neisseria* are seen, as too are those containing both *Streptococcus* and *Haemophilus*.
