# Supplementary material for: The upper respiratory tract microbiome of indigenous Orang Asli in north-eastern Peninsular Malaysia
Source: NPJ Biofilms Microbiomes. 2021 Jan 5;7:1. doi: 10.1038/s41522-020-00173-5 (PMC7785749; doi:10.1038/s41522-020-00173-5)
Supplement: Supplementary file 2 — Reporting Summary Checklist [file 41522_2020_173_MOESM2_ESM.pdf]

nature research

Corresponding author(s): David William Cleary

Last updated by author(s): Nov 10, 2020

Reporting Summary

Nature Research wishes to improve the reproducibility of the work that we publish. This form provides structure for consistency and transparency in reporting. For further information on Nature Research policies, see our Editorial Policies and the Editorial Policy Checklist.

Statistics

For all statistical analyses, confirm that the following items are present in the figure legend, table legend, main text, or Methods section.

n/a	Confirmed	
☐	☒	The exact sample size (n) for each experimental group/condition, given as a discrete number and unit of measurement
☐	☒	A statement on whether measurements were taken from distinct samples or whether the same sample was measured repeatedly
☐	☒	The statistical test(s) used AND whether they are one- or two-sided<br>*Only common tests should be described solely by name; describe more complex techniques in the Methods section.*
☐	☒	A description of all covariates tested
☐	☒	A description of any assumptions or corrections, such as tests of normality and adjustment for multiple comparisons
☐	☒	A full description of the statistical parameters including central tendency (e.g. means) or other basic estimates (e.g. regression coefficient) AND variation (e.g. standard deviation) or associated estimates of uncertainty (e.g. confidence intervals)
☐	☒	For null hypothesis testing, the test statistic (e.g. F, t, r) with confidence intervals, effect sizes, degrees of freedom and P value noted<br>*Give P values as exact values whenever suitable.*
☒	☐	For Bayesian analysis, information on the choice of priors and Markov chain Monte Carlo settings
☒	☐	For hierarchical and complex designs, identification of the appropriate level for tests and full reporting of outcomes
☒	☐	Estimates of effect sizes (e.g. Cohen's d, Pearson's r), indicating how they were calculated

Our web collection on statistics for biologists contains articles on many of the points above.

Software and code

Policy information about availability of computer code

Data collection	No software was used.
Data analysis	Microbiome Analysis:<br><br>Initial data handling was done using QIIME 2 2018.8.<br>Raw sequence data were denoised with DADA2.<br>Amplicon sequence variants (ASVs) were aligned with mafft (via q2-alignment) and a phylogeny constructed using fasttree2.<br>Taxonomic classification was done using the q2-feature-classifier classify-sklearn naïve Bayes taxonomy classifier using the Greengenes 13_8 (99%) reference sequences.<br>The feature table, taxonomy, phylogenetic tree and sample metadata were then combined into a Phyloseq object using qiime2R via qza_to_phyloseq.<br>All further analysis was done in R v3.6.0 73 in RStudio and figures were produced using the package ggplot2.<br>Phyloseq v1.29.0 was used following a workflow previously described.<br>Potential contaminants were identified removed using the R package 'decontam'.<br>Differentially abundant ASVs were identified using the DESeq2 package.<br>Networks were generated using the R implementation of SPIEC-EASI (SpiecEasi) with results visualised using ggnet().

For manuscripts utilizing custom algorithms or software that are central to the research but not yet described in published literature, software must be made available to editors and reviewers. We strongly encourage code deposition in a community repository (e.g. GitHub). See the Nature Research guidelines for submitting code & software for further information.

Data

Policy information about availability of data

All manuscripts must include a data availability statement. This statement should provide the following information, where applicable:

- Accession codes, unique identifiers, or web links for publicly available datasets
- A list of figures that have associated raw data
- A description of any restrictions on data availability

Sequence files and associated metadata have been deposited in the European Nucleotide Archive (ENA) in project PRJEB38610 under experiment accessions ERX4147052 to ERX4147288. Code is available at: https://github.com/davidwcleary/orang_asli_16S/blob/master/OrangAsli.Rmd

Field-specific reporting

Please select the one below that is the best fit for your research. If you are not sure, read the appropriate sections before making your selection.

☒ Life sciences ☐ Behavioural & social sciences ☐ Ecological, evolutionary & environmental sciences

For a reference copy of the document with all sections, see nature.com/documents/nr-reporting-summary-flat.pdf

Life sciences study design

All studies must disclose on these points even when the disclosure is negative.

Sample size	This was a pragmatic, all-age observational carriage study. Consequently it was not possible to undertake any sample size calculations.
Data exclusions	No data was excluded.
Replication	No replication was done.
Randomization	Not relevant.
Blinding	Participants were given annonymised sample reference numbers to which researchers were blinded.

Reporting for specific materials, systems and methods

We require information from authors about some types of materials, experimental systems and methods used in many studies. Here, indicate whether each material, system or method listed is relevant to your study. If you are not sure if a list item applies to your research, read the appropriate section before selecting a response.

Materials & experimental systems

n/a	Involved in the study	
☒	☐	Antibodies
☒	☐	Eukaryotic cell lines
☒	☐	Palaeontology and archaeology
☒	☐	Animals and other organisms
☐	☒	Human research participants
☒	☐	Clinical data
☒	☐	Dual use research of concern

Methods

n/a	Involved in the study	
☒	☐	ChIP-seq
☒	☐	Flow cytometry
☒	☐	MRI-based neuroimaging

Human research participants

Policy information about studies involving human research participants

Population characteristics

Kampung Su
ngai Pergam (Site 1) Kampung Berua (Site 2) p value
Total (n) 68 62

Gender
Female 34 30
Male 32 26
NA 2 6

Household Size (Average, n) 4.5 5.2

Age (years)
<5 1 6
5 to <18 27 39
18 to <65 25 12
65+ 1 0
NA 14 5 <0.05

Vaccinations
Yes 61 50
No 5 4
NA 2 8

Smoking
Yes 14 7
No 53 51
NA 1 4

Respiratory Symptoms
Within last 30 days 40 16
Within last 90 days 36 6

Questionnaires were used to capture participant metadata which included gender, age, number of dwelling co-occupants and occupation in addition to health-related questions including current or recent (within the last month and last three months) respiratory symptoms, antibiotic use and vaccination status.

Recruitment	Two Orang Asli villages were visited in August 2017 – Kampung Sungai Pergam in Kemaman district on the 8th August, and Kampung Berua in Hulu Terengganu district on the 9th August. Both sites are located in the state of Terengganu which lies in the north-east of Peninsular Malaysia. Participant recruitment was with consent, across all ages with no exclusion criteria.
Ethics oversight	Ethical approval for this study was provided by Universiti Sultan Zainal Abidin (UniSZA) Ethics Committee: approval no. UniSZA/C/1/UHREC/628-1(85) dated 27th June 2016, the Department of Orang Asli Affairs and Development (JAKOA): approval no. JAKOA/PP.30.052Jld11 (42), and by the University of Southampton Faculty of Medicine Ethics Committee (Submission ID: 20831).

Note that full information on the approval of the study protocol must also be provided in the manuscript.
